# Completing the Puzzle: Determinants, Comorbidities and Complications for Different Lung Cancer Subtypes: A Pilot Study

**DOI:** 10.3390/life14121611

**Published:** 2024-12-05

**Authors:** Corina Eugenia Budin, Iuliu Gabriel Cocuz, Adrian-Horațiu Sabău, Raluca Niculescu, Cristian Cazacu, Edith-Simona Ianoși, Ovidiu Simion Cotoi

**Affiliations:** 1 Pathophysiology Department, George Emil Palade University of Medicine, Pharmacy, Science and Technology of Târgu Mureș, 540139 Targu Mures, Romania; corina.budin@umfst.ro (C.E.B.); adrian-horatiu.sabau@umfst.ro (A.-H.S.); raluca.niculescu@umfst.ro (R.N.); ovidiu.cotoi@umfst.ro (O.S.C.); 2 Pneumology Department, Mures Clinical County Hospital, 547530 Targu Mures, Romania; edith.ianosi@umfst.ro; 3 Pathology Department, Mures Clinical County Hospital, 547530 Targu Mures, Romania; 4 Faculty of Medicine, George Emil Palade University of Medicine, Pharmacy, Science and Technology of Târgu Mureș, 540139 Targu Mures, Romania; cazacu.cristian99@yahoo.com; 5 Pneumology Department, George Emil Palade University of Medicine, Pharmacy, Science and Technology of Târgu Mureș, 540139 Targu Mures, Romania

**Keywords:** lung cancer, comorbidities, smoking

## Abstract

Background: Lung cancer remains one of the leading causes of cancer-related mortality worldwide, with multiple independent risk factors contributing to its development. The objective of this study was represented by the impact of independent risk factors, such as smoking, anemia, cachexia or COPD (chronic obstructive pulmonary disease) for lung cancer development. Methods: We conducted a retrospective study, and we analyzed a database of 412 patients hospitalized between 1 February and 31 December 2023 in the Pulmonology Department of the Mureș County Clinical Hospital. Following the analysis of the inclusion and exclusion criteria, the final analyzed group included 115 patients. Results: From the study group, 88 patients were diagnosed with non-small cell lung cancer and 27 with small cell lung cancer. Of the non-small cell lung cancer patients, 50% had adenocarcinoma and 50% had squamous cell carcinoma. Chronic obstructive pulmonary disease and cardiovascular diseases predominate as concomitant pathologies, with 82 and 81 cases identified among the patients evaluated, respectively. The incidence of diabetes mellitus was n = 20 for the patients, followed by asthma and other neoplasms. The body mass index was also analyzed with an average of 24.6. Body mass index does not correlate with histological type. The mean hemoglobin value in the group of patients was 12.8, and this could not be correlated with the histopathological type. Conclusions: Chronic obstructive pulmonary disease and lung cancer may just be two different clinical presentations based on the same etiological factors, which also have a lot of overlapping pathophysiological mechanisms. Therefore, Chronic obstructive pulmonary disease represents an individual risk factor for developing lung cancer. Smoking, as well as anemia, cachexia or other comorbidities (COPD), are individual risk factors for lung cancer.

## 1. Introduction

The main and most important risk factor for lung cancer is smoking. It is estimated that 75–80% of all lung cancer deaths are directly related to smoking [1]. Cigarette smoke contains a multitude of both organic and inorganic carcinogens, which include polycyclic aromatic hydrocarbons (PAHs), aromatic amines, N-nitrosamines, benzene, vinyl chloride, arsenic, chromium and more [2]. The risk of developing lung cancer is directly related to the degree of exposure to cigarette smoke, varying directly proportional to the inhaled dose. The degree of exposure to smoke depends on factors such as the type of cigarette, the duration of inhalation and the presence of a filter [3]. Smoking is more prevalent among men, with 27% of them smoking compared to 23% of women. The risk of lung cancer is strongly associated with the number of cigarettes smoked per day and the duration of smoking [4].

Although smoking is linked to all types of lung cancer, its most significant associations are with small cell lung cancer (SCLC) and squamous cell lung cancer [3]. Malignant transformation of other chronic pulmonary pathologies is also a scientifically verified hypothesis. Thus, diseases such as chronic obstructive pulmonary disease (COPD) and idiopathic pulmonary fibrosis (IPF) maintain a state of chronic inflammation in the lungs. Persistent inflammation in these conditions can cause DNA damage in lung cells, making them more susceptible to mutations and malignant transformations. Therefore, patients with these conditions face a high risk of developing lung cancer [5]. Nutritional status is an independent and additional risk factor for lung cancer progression and staging. For lung cancer patients, patients’ body mass index (BMI) has two different extremely important roles, being associated with both the risk of developing lung cancer and overall survival if it has already been diagnosed.

In general, it is known that obesity is an independent risk factor for developing various neoplasms, with BMI thus directly influencing the risk of developing cancer. However, this correlation does not seem to be valid in the case of NSCLC lung cancer, where the opposite has been observed; namely, that an increased BMI is protective [6]. The cause of anemia in cancer patients has not been fully elucidated, as it is multifactorial. Thus, it is assumed that in addition to chemotherapy and radiotherapy, which may have a direct impact on hematopoiesis, tumor cells can produce various cytokines that interfere with the production of erythrocytes, thus leading to a decrease in their number. Regardless of the cause of anemia, its presence is an independent factor for unfavorable prognosis in the overall survival of patients. This association remains valid in all patient groups, regardless of age, gender or stage of the disease [7]. The stated objective of this study was represented by the impact of associated pathologies such as anemia, cachexia or COPD in patients with histopathological confirmed lung cancer, as well as the correlation of these parameters with lung cancer subtypes.

## 2. Materials and Methods

To carry out this research, we conducted a retrospective study by collecting the necessary information from the information system of the Mureș County Clinical Hospital, Pulmonology Clinic, from the integrated information system Hipocrates version H3 concept. The data collected were from 1 February 2023 to 31 December 2023. Access to the hospital’s archive to consult observation sheets was granted by the Management of the Hospital and received the approval of the Ethics Commission with number 6089/28.05.24. This study was carried out in accordance with the principles set out by the Declaration of Helsinki.

The information collected was as follows: gender; age; primary diagnosis; secondary diagnostics; associated pathologies; survival status; laboratory tests and inflammatory markers; histopathological type (NSCLC, SCLC); and molecular mutations in those with NSCLC. Inclusion criteria were represented by the following: patients hospitalized in continuous hospitalization in the Pulmonology department with a confirmed diagnosis of bronchopulmonary neoplasm by histopathological examination on the biopsy sample; adult patients; and those over 18 years of age. Exclusion criteria were represented by patients with clinical or imaging suspicion of lung cancer, but in whom the histopathological examination was negative for malignancy; patients for whom the results of investigations (blood tests, molecular mutations) were not available; and patients under 18 years of age. A database of 412 patients hospitalized between 1 February and 31 December 2023 in the Pulmonology Department of the Mureș County Clinical Hospital was analyzed. Following the analysis of the inclusion and exclusion criteria, the final analyzed group included a count of 115 patients. The study was carried out in accordance with and in compliance with the confidentiality clauses regarding the processing of personal data, with the data extracted from the computer system of the County Clinical Hospital of Târgu Mureș being used exclusively for the purpose of conducting this study.

### Statistical Analysis

The data were centralized in Microsoft Excel spreadsheets. Statistical analysis was performed in the R statistical system (version 4.3.3, released on 29 February 2024). Continuous variables were checked for Gaussian distribution using the Shapiro–Wilk test. Comparisons between groups in terms of numerical variables were performed using the Wilcoxon or Kruskal–Wallis tests. Categorical variables were compared using Fisher’s exact test for counting data. Before fitting several logistic regression models, we performed an analysis of the missing data, followed by multiple imputations by chained equations. Over the course of the study, a statistical significance threshold of 0.05 was used.

## 3. Results

**Figure 1:**
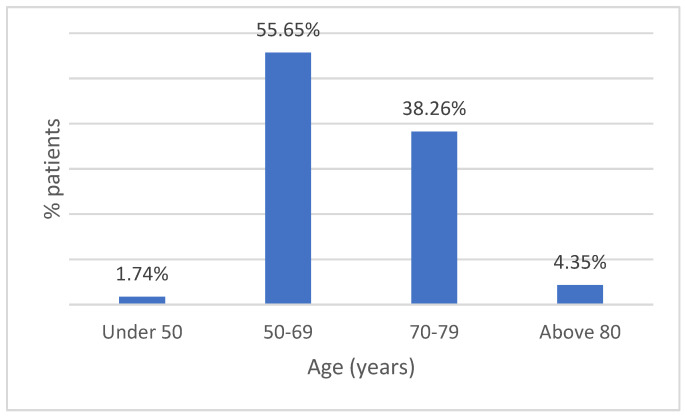
Age distribution of patients diagnosed with lung cancer.

**Figure 2:**
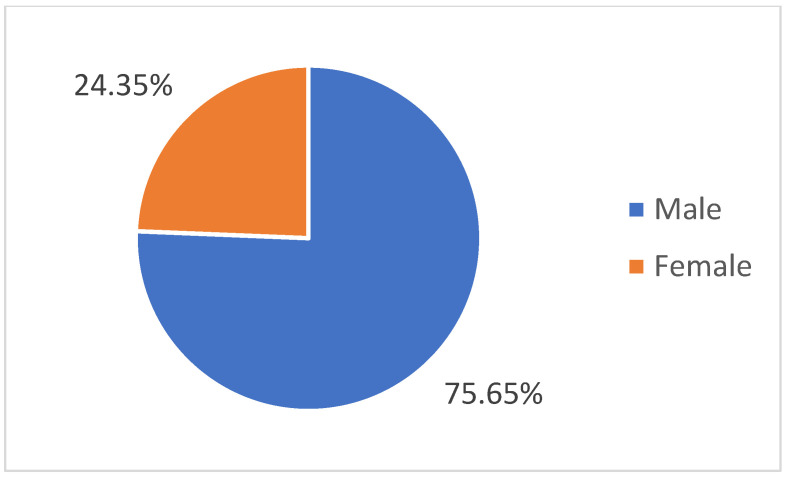
Gender distribution of patients diagnosed with lung cancer.

In this retrospective study, we analyzed a total of 115 patients, with a mean age of 68 years and age distribution represented in Figure 1, of which 24.3% were female and 75.7% were male, respectively (Figure 2).

**Figure 3:**
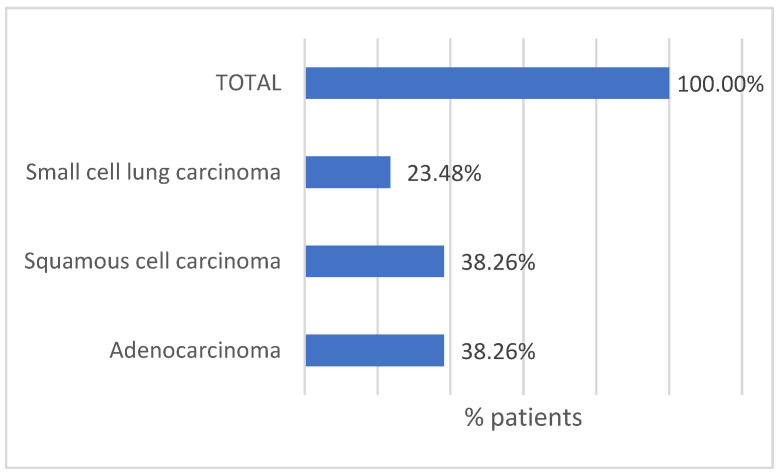
Histopathological subtype of malignancies in patients diagnosed with lung cancer.

The histopathological diagnosis was established on biopsy specimens. Patients were histopathological diagnosed with “non-small cell lung carcinoma” (NSCLC) or “small cell lung cancer” (SCLC). The NSCL was subdivided into squamous cell carcinoma and adenocarcinoma, as presented in Figure 3.

Of the 88 patients with NSCLC, molecular mutation data were available in 69 patients. Of the patients for whom molecular mutations were available, 7 had the EGFR mutation present, 1 patient had the ALK mutation and 25 patients had alterations in PD-L1 expression influencing PD-L1 regulation. The type of NSCLC and the diagnosed mutation can be seen in Table 1.

**Figure 4:**
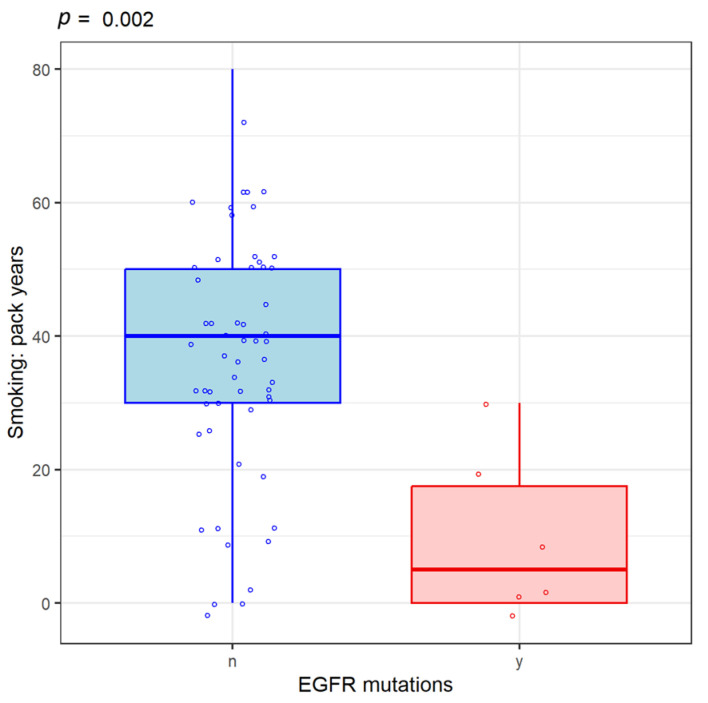
Correlation between smoking and EGFR mutations. y (yes) = Presence of EGFR mutations. n (no) = Absence of EGFR mutations.

Age does not correlate with histological type (*p* = 0.17), EGFR mutations (*p* = 0.94) or PD-L1 expression (*p* = 0.75). In patients with EGFR mutations, the most common histological type was adenocarcinoma (*p* = 0.04). The number of cigarette packs smoked by those without EGFR mutations was higher than in those with EGFR mutations (*p* = 0.002). (Figure 4). The average hemoglobin value for the patients included in the study was 12.8 mg/dL and could not be correlated with the histopathological type of NSCLC.

**Figure 5:**
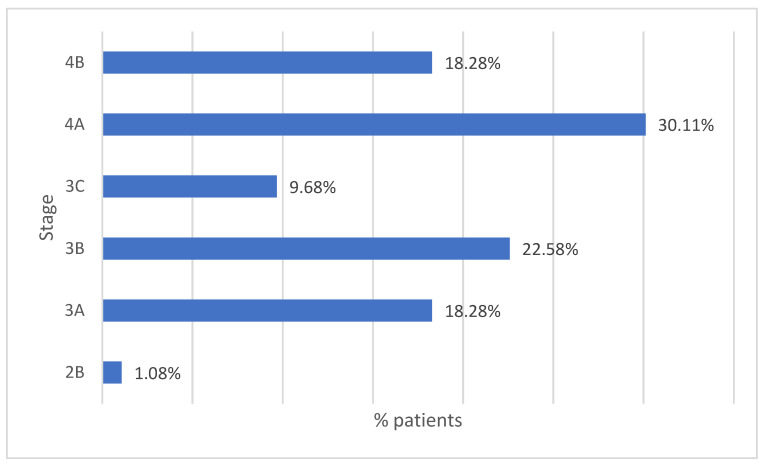
Distribution of patients according to disease stage at first presentation.

Of the total group of 115 patients, only 93 were accurately diagnosed with the stage of the disease upon admission. Analyzing the distribution of patients according to the stage of the disease at the first presentation, we can see that the smallest number of patients presented stage 2B (n = 1). Stages 3A and 3B showed relatively similar frequencies, with 17 and 21 patients diagnosed, respectively. Stage 3C was identified in nine patients, showing a lower incidence of cancer diagnosis frequency at this stage. It is important to note that most patients were diagnosed in stage 4A (n = 28), followed by stage 4B (n = 17), which once again emphasizes the presentation of patients in the advanced stage of the disease (Figure 5).

Chronic obstructive pulmonary disease (COPD) and cardiovascular diseases predominate as concomitant pathologies with 82 and 81 cases identified among the patients evaluated, respectively. The incidence of diabetes mellitus in 20 of the patients, followed by asthma and other neoplasms (with 12 and 11 cases, respectively), indicates the diversity of associated pathologies that can influence the clinical management and prognosis of patients. COPD was more common in patients without the EGFR mutation than in those with EGFR mutations (*p* = 0.002).

**Figure 6:**
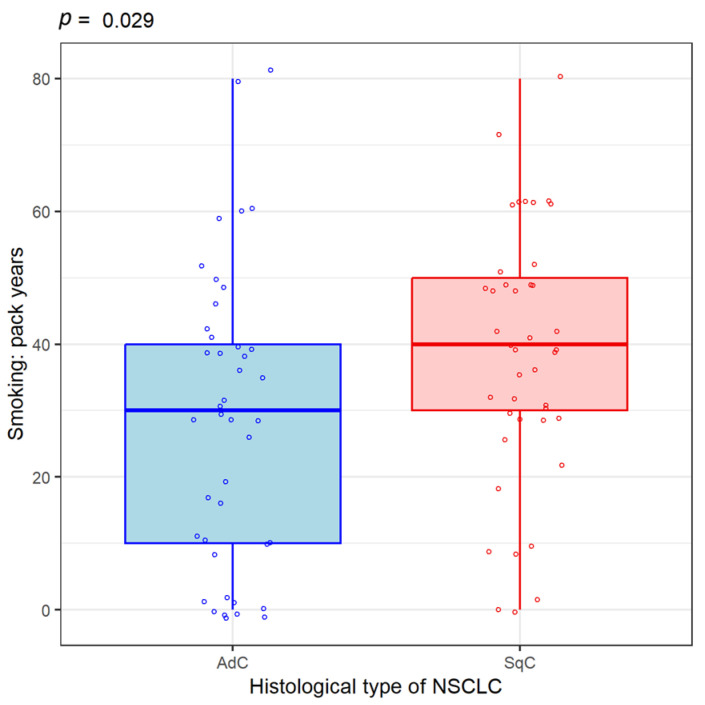
Association of smoking with the histopathological type of lung cancer. AdC = adenocarcinoma. SqC = Squamous cell carcinoma. NSCLC = non-small cell lung carcinoma.

**Figure 7:**
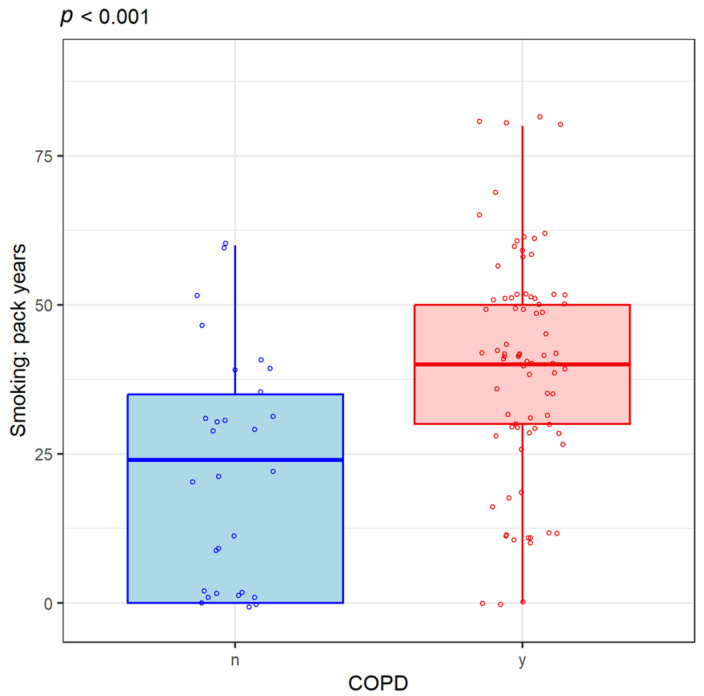
Frequency of smoking in lung cancer and COPD patients compared to those with lung cancer without a diagnosis of COPD. y (yes) = Presence of COPD. n (no) = Absence of COPD.

Other data collected from patients included the number of cigarettes consumed daily and how long they were smokers; this information was expressed as a “pack-year” index. Thus, the patients included in the study had an average of 40 packs per year. We correlated the histological type of lung cancer with smoking to see if it disproportionately increases the risk of a particular malignancy subtype. Thus, the number of packs/year was higher in patients with squamous cell carcinoma than in those with adenocarcinoma (Figure 6). Among lung cancer patients, those with concomitant COPD diagnoses smoked more on average than those with lung cancer without a diagnosis of COPD (Figure 7). The presence of COPD is associated with the histological type of lung cancer: there are more cases of squamous cell carcinoma (SqC) and SCLC in patients with COPD than in those without COPD.

Body mass index (BMI) was also analyzed, with an average of 24.6 being found for the 115 patients, with a range between 21.4 and 27.3. Body mass index (BMI) does not correlate with histological type.

## 4. Discussion

SCLC is found to have 250,000 new cases and cause at least 200,000 deaths annually worldwide. The prevalence of lung cancer, which encompasses all histological subtypes, is higher in high-income countries and regions, and is linked to varying rates of tobacco use [8]. Over the past four decades, there has been a notable decline in the relative occurrence of SCLC. This decline can be attributed to a decrease in smoking, changes in the composition of cigarettes and a decrease in the risks of occupational exposure. Historically, SCLC has shown a higher incidence among men. However, the incidence gap between men and women has narrowed considerably over the past three decades, explained by the relative increase in smoking among women [3]. According to scientific studies, there is a delay in presenting in patients, who are later diagnosed with lung cancer by medical services [9]. Our study found that participants tended to have a significant history of smoking exposure, measured at 40 pack/years (PY), which is consistent with the data gathered. This metric highlights smoking’s significance as a risk factor in our population by reflecting the cumulative effects of smoking over time. Furthermore, our results show significant differences between participants based on the non-small cell lung cancer (NSCLC) subtype. This distinction by NSCLC subtype raises the possibility that smoking exposure may affect both the risk of acquiring lung cancer and the subtype that appears. These findings highlight how crucial it is to assess and manage lung cancer risk by considering both smoking history and cancer subtype. Another problem that delayed diagnoses was the presence of comorbidities. Thus, many of the new symptoms that appeared were not seen as a new entity but were explained by the presence of chronic pathologies that the patient has.

Multiple studies have observed this paradoxical association, but their limitation was the presence of smoking in the group of people studied, which is a confounding factor that influences both BMI (decreases it) and the risk of lung cancer (increases it) [10].

Thus, in 2018, a study conducted on 1.6 million patients managed to demonstrate that a high BMI is indeed an independent protective factor for developing NSCLC lung cancer, but having a positive association with the risk of developing SCLC. The cause of both the unexpected impact of obesity on lung cancer compared to other neoplasms, as well as the different influence it has depending on the cancer subtype (SCLC vs. NSCLC), remains unknown. It is worth mentioning that although BMI is a protective factor for NSCLC, central obesity, regardless of the patient’s BMI, expressed as waist circumference or waist-to-hip ratio, is associated with an increased risk of all forms of lung cancer (with an increase of every 10 cm in abdominal circumference leading to a 10% increase in the risk of lung cancer), thus maintaining the directly proportional relationship described for other neoplasms [11].

At the same time, BMI is a prognostic factor for overall survival (OS) in lung cancer patients. BMI variation, described as its variation between early maturity and the date of diagnosis, is associated with OS; i.e., a lower BMI value at the time of diagnosis compared to the one the patient had in youth correlates with a poor prognosis. A U-shaped association between BMI at diagnosis and overall survival (OS) has also been reported, with high mortality observed in the extreme-case groups of patients (underweight and morbidly obese) compared to normal-weight patients, while the best OS outcomes were recorded in overweight or obese individuals (but not morbidly obese), highlighting once again a possible protective effect of the presence of obesity against both the genesis and the evolution of lung cancer [12]. We were unable to adequately evaluate overall survival, a crucial indicator of therapy effectiveness and long-term results, due to the short patient follow-up period in this investigation. Therefore, this brief follow-up period is a drawback since it limits our capacity to completely comprehend the effects of the interventions under study on survival. Future research with longer follow-up is necessary to offer a thorough assessment of overall survival because it is difficult to capture the long-term advantages or dangers of the treatment without a prolonged observation period. In the scientific literature, it is described that most lung cancer patients have a normal BMI or are overweight at the time of diagnosis; those with extreme values are very few. In the present study, the mean BMI at diagnosis of the 115 patients confirms once again the data presented in the literature. In this study, the mean BMI of the patients was 24.6, and the attempt to statistically correlate BMI with the histopathological type of cancer did not reveal a significant association, concluding that BMI does not correlate with the cancer subtype. However, it must be considered that one of the limitations of the present study is the small group of patients; therefore, this correlation could be investigated in future studies with more patients included, with the current study being a pilot study with possibilities for further expansion.

Anemia is frequently observed in neoplastic patients; in some studies, it is found in about 30% of patients. It is also a common complication in lung cancer patients. In the study conducted by Aoe et al., 48.8% of patients had anemia at the time of diagnosis, and other studies have shown that it occurs in most patients at some point in the course of the disease [13]. To date, studies have not been able to demonstrate a correlation between anemia and the histological subtype of cancer, which is present in different degrees in all patient groups [7]. Although in our study the hemoglobin value was 12.8 mg/dL and could not be correlated with the histopathological type, this does not exclude the fact that there may be an association, but, to discover it a meta-analysis is needed on a large number of patients.

The main and most important risk factor for lung cancer is smoking [14]. Importantly, smoking disproportionately increases the risk of certain histological types of lung cancer. Thus, multiple studies have confirmed that adenocarcinoma is the most common type of lung cancer found in women and non-smokers, while the frequency of squamous cell cancer and SCLC related to adenocarcinoma increases directly proportionally to the BP index that quantifies smoking [15]. This fact may be related to the squamous metaplasia that appears in smoking related patients, the starting point of dysplasia in many patients diagnosed with squamous cell carcinoma of the lung. The results of this study once again emphasize this idea by the fact that in the group of patients, statistically, the number of packs/year was higher in patients with squamous cell carcinoma than in those with adenocarcinoma.

COPD is an independent risk factor for developing lung cancer. This fact has been a controversial topic for a long time, because most of the etiological agents that contribute to the development of COPD also play a key role in the genesis of lung cancer. Most patients with COPD and lung cancer have smoking as their main confounding factor [16]. In a recent study from 2021, it was shown that there is a significant association between COPD history and lung cancer risk in various subgroups such as the following: men, women, non-smokers, current smokers, former smokers. It concluded that those who are diagnosed with COPD have at least twice the risk of developing lung cancer than those without COPD. The same positive association was found in the stratified analysis depending on the predominant involvement of COPD: chronic bronchitis and emphysema, respectively.

Smokers and non-smokers were all at risk for lung cancer, but emphysema was linked to a higher risk of carcinogenesis than chronic bronchitis [17]. Another study was able to demonstrate that both the severity of bronchial obstruction and the frequency of acute respiratory exacerbations are independent risk factors for lung cancer [18]. COPD significantly increases the risk in all histological types of lung cancer, but a closer correlation was observed between predominantly emphysema COPD and squamous cell carcinoma, respectively. The incidence of adenocarcinoma is more likely to occur in COPD that is primarily of the chronic bronchitis type than other subtypes [19,20]. In the current study, the results showed that patients with lung cancer and COPD smoked more than those without COPD on average, consistent with information in the literature that supports that smoking contributes to the development of both pathologies. We have also demonstrated once again that the presence of COPD is associated with an increased risk of developing mostly squamous cell carcinoma or SCLC [21].

## 5. Conclusions

Measuring the level of tobacco dependence may be a presumptive factor for assessing histological subtypes and molecular mutations in lung cancer, in addition to the predictive value of smoking as an independent risk factor for the disease. An evaluation of mortality and, consequently, overall survival (OS) may be made easier by extending the follow-up duration for this population. Additional information on other assessed parameters might be obtained by conducting follow-ups at six and twelve months. Hemoglobin levels and BMI, for instance, may change over time due to the neoplastic disease itself as well as directly due to therapy adverse effects. Examining these lung cancer determinants concurrently could result in a more precise diagnostic framework that includes possible predictors for histopathological diagnosis.

## Figures and Tables

**Table 1:** Type of NSCLC and available mutations in patients diagnosed with lung cancer.

NSCL Subtype	Performed (n)	Negative (n)	EGFR (n)		ALK (n)		PDL-1 (IHC) (n)		KRAS (exon 2) (n)
			+	NI	+	NI	+	NI	+
Adenocarcinoma	33	12	6	2	1	1	13	2	1
Scuamous cell carcinoma	36	24	1	-	0	-	12	-	-
Total	69	36	7	2	1	1	25	2	1

Legend: NI—not interpretable, “+” = Presence of the mutation, “-” = Not the case, EGFR—Activating mutations in exons 18, 19, 20 and 21 of the EGFR gene, ALK—driver fusions in the ALK gene

## Data Availability

The original contributions presented in this study are included in the article. Further inquiries can be directed to the corresponding author.
